# The Impact of Manuscript Learning vs. Video Learning on a Surgeon's Confidence in Performing a Difficult Procedure

**DOI:** 10.3389/fsurg.2018.00067

**Published:** 2018-11-12

**Authors:** Carlos A. Reck-Burneo, Alexander J. M. Dingemans, Victoria A. Lane, Jennifer Cooper, Marc A. Levitt, Richard J. Wood

**Affiliations:** ^1^Center for Colorectal and Pelvic Reconstruction (CCPR), Nationwide Children's Hospital, Columbus, OH, United States; ^2^Department of Surgery, Medical University of Vienna, Vienna, Austria

**Keywords:** pediatric surgery, medical education, operative videos, manuscript, learning surgery

## Abstract

**Introduction:** Operative surgical videos are a popular educational resource, not commonly a part of a peer-reviewed article. We wanted to evaluate the impact of either reading a peer-reviewed manuscript or watching an operative video on a surgeon's confidence in performing a complex case.

**Methods:** Pediatric surgeons and fellows were asked to complete an initial questionnaire to assess their confidence (formulated as a score) in the diagnosis and operative repair of anal stenosis and rectal atresia.

**Results:** Of 101 pediatric surgeons and fellows, 52 (51%) were randomized into a “manuscript” group and 49 (49%) into a “video” group. The mean confidence before the intervention was the same in the two groups (6.4 vs. 6.6). Attending surgeons started with more confidence than trainees (7.1 vs. 5.3, *p* < 0.001). In the manuscript group, the average confidence increased to 7.7 (*p* = 0.005), and in the video group the average confidence increased to 7.9 (*p* = 0.001) globally. Trainees in the video group significantly improved their confidence to a score of 6.6 (*p* = 0.035), as did attending surgeons to 8.5 (*p* = 0.01). In the manuscript group, only attendings significantly improved their confidence by 1.5–8.3 (*p* < 0.001), whereas trainees did not with a difference of 1.3 (*p* = 0.194). When considering experience level, physicians who reported never having performed this surgery improved only by reading the manuscript (3.9–6.2) (*p* = 0.004), not by watching the video (5.4–6.6) (*p* = 0.106). Surgeons with experience doing this operation (>5 times) did not improve their confidence by reading the manuscript (*p* = 0.10), nor by watching the video (*p* = 0.112).

**Conclusion:** Reviewing either a detailed manuscript or operative video on the surgical management of rectal atresia and anal stenosis demonstrated a significant increase in self-reported confidence. Trainees benefitted the most from operative videos, whereas experienced surgeons did not improve their confidence by reading the manuscript nor watching the video.

## Introduction

Traditionally, surgeons and residents have learned new operative techniques by reading about surgical techniques and trying to improve their skills in the operating room. Recently, newly emerged media, such as video, are already being used in educating medical students and seem to have value as an addition to traditional educational measures (1, 2). In recent years, several services have surfaced which provide videos of surgeries as a way of teaching the audience a specific surgical technique. There is, however, little evidence that the use of these videos is an improvement over the use of a manuscript. Therefore, we set out to compare if watching a peer-reviewed video improves the confidence of surgeons in performing an operation more than the reading of a peer-reviewed technical manuscript.

## Methods

### Inclusion/exclusion criteria

Any pediatric surgeon or pediatric surgery resident could participate in this study. The study was approved and excempted from a full review by the Institutional Review Board of Nationwide Childrens Hospital due to the voluntary, consented and anonymous participation of our study population. We contacted surgeons and residents by email and asked them to anonymously complete a questionnaire assessing their confidence in diagnosing and operating on patients with anal stenosis or rectal atresia. The participants were then randomized into two groups: one group watched the video, and the other group read the manuscript. Thereafter, they completed the same questionnaire as before the intervention, to assess any difference in confidence.

### Instruments used

To assess the confidence of physicians in performing the surgery, we developed an 11-item questionnaire (see addendum A). Participants were asked about their experience in these operations and whether they were a trainee or an attending surgeon, enabling us to perform a subgroup analysis on these groups. For the overall analysis, we looked at the individual questions, but averaged the confidences of the questions into a total score as well.

The manuscript describing the operative technique was published earlier in a peer-reviewed journal (3). Furthermore, the technique video on the same topic was also peer-reviewed and published by the same team on the C-Surgeries video site.

### Statistics

Mann-Whitney *U*-tests were performed to compare the total pre- and post-confidence between the video and the manuscript groups. The same test was used to see if there was any improvement in either group for the subgroup analysis and to look at the individual questions. For the overall comparison, a *p* < 0.05 was considered significant. When comparing the individual questions, after applying a Bonferroni correction, a *p* < 0.00833 was considered significant.

## Results

### Demographics

One hundred one surgeons completed the pre- and post-intervention questionnaires. Of those, 52 (51%) were randomized to the manuscript group and 49 (49%) to the video group. Of the surgeons completing both surveys, 66 (65%) were attending pediatric surgeons, 19 (65%) were pediatric surgery residents (19%), and 16 (16%) were pediatric surgery fellows. Thirty-two (32%) participants reported never having performed either surgery, whereas 46 (46%) participants had performed it between one to five times, and 23 (23%) surgeons had performed the surgery more than five times.

### Pre-intervention confidence

The baseline overall confidence was 6.6 in the video group and 6.4 in the manuscript group (*p* = 0.962). Attending surgeons reported an overall confidence of 7.4 in the video group and 6.8 in the manuscript group, whereas these numbers were 5.2 and 5.5 for trainees. Physicians who had not previously performed the surgery noted a pre-intervention confidence of 5.4 in the video group and 3.9 in the manuscript group, whereas these were 6.4 and 6.7 for surgeons having performed this surgery one to five times. Finally, surgeons that had performed this particular technique at least five times reported a confidence of 8.7 in the video group and 8.5 in the manuscript group before receiving the intervention.

### Post-intervention confidence

After either reading the manuscript or watching the video, the confidence rose to 7.7 (*p* = 0.005) in the video group and to 7.9 (*p* = 0.001) in the manuscript group. When comparing the two groups, there was no significant difference between the total scores (*p* = 0.790) or any of the separate questions. However, when looking at the individual questions in the video and manuscript groups separately, the manuscript group improved significantly on five of six questions, whereas the video group improved only on two questions (see Table 1).

**Table 1:** All questions an the average confidence score per group.

**Question**	**Video baseline**	**Video post-intervention**	***P*-value**	**Paper baseline**	**Paper post-intervention**	***P*-value**
How confident on a scale of 1-10 are you in being able to clinically diagnose a child with rectal atresia or anal stenosis?	7.8	7.9	0.666	7.6	8.5	0.015
How confident are you in your knowledge of the anatomical difference between rectal atresia and anal stenosis?	7.3	8.1	0.076	7.3	8.6	0.002^*^
How confident are you in being able to describe the technique for the repair of rectal atresia on a scale of 1-10?	6.3	8	< 0.001^*^	6.2	7.8	0.002^*^
How confident are you in being able to describe the technique for the repair of anal stenosis.	6.5	8.1	0.001^*^	6.1	7.8	< 0.001^*^
How confident are you in performing the surgery for rectal atresia (with preservation of the anal canal)?	5.8	7	0.013	5.8	7.3	0.003^*^
How confident are you in performing the surgery for anal stenosis (with preservation of the anal canal)?	5.8	7	0.012	5.6	7.3	0.001^*^

* *Significant after applying Bonferroni correction*.

### Subgroup analysis (trainee/attending, times performed)

We also analyzed the influence of experience on the increase of confidence (see Table 2). When shown the video, trainees improved their confidence from 5.2 (*n* = 26) to 6.6 (*n* = 20, *p* = 0.035). The confidence of attending surgeons who watched the educational video increased from 7.4 (*n* = 43) to 8.5 (*n* = 29, *p* = 0.01).

**Table 2:** Experience and the influence on confidence.

**Video baseline**		**Video after**		***P*-value**
**PREVIOUSLY PERFORMED THIS SURGERY**				
Never (*n* = 23)	5.4	Never (*n* = 18)	6.6	0.106
1–5 times (*n* = 31)	6.4	1–5 times (*n* = 21)	7.8	0.006
>5 times (*n* = 15)	8.7	>5 times (*n* = 10)	9.5	0.09
**OVERALL EXPERIENCE**				
Trainee (*n* = 26)	5.2	Trainee (*n* = 20)	6.6	0.035
Attending (*n* = 43)	7.4	Attending (*n* = 29)	8.5	0.01
**Paper baseline**		**Paper after**		
**PREVIOUSLY PERFORMED THIS SURGERY**				
Never (*n* = 25)	3.9	Never (*n* = 14)	6.2	0.004
1–5 times (*n* = 32)	6.7	1–5 times (*n* = 25)	8.2	0.001
>5 times (*n* = 27)	8.5	>5 times (*n* = 13)	9.1	0.112
**OVERALL EXPERIENCE**				
Trainee (*n* = 24)	5.5	Trainee (*n* = 15)	6.8	0.194
Attending (*n* = 60)	6.8	Attending (*n* = 37)	8.3	0.001

Attending surgeons who read the manuscript significantly increased their scores, as well (from 6.8 to 8.3, *p* = 0.001). This was not the case for trainees: as a result of reading the article, there was no statistically significant increase in confidence (5.5–6.8, *p* = 0.194).

We also took into account the number of times a surgeon had done this particular operation. Surgeons having never performed this operation significantly improved their confidence in both groups: from 5.4 to 6.6 (*p* = 0.048) in the video group and from 3.9 to 6.2 (*p* < 0.001) in the manuscript group. The same conclusion can be drawn when looking at surgeons who had performed this surgery between one and five times: when watching the video, their confidence rose from 6.4 to 7.8 (*p* = 0.006), whereas it increased from 6.7 to 8.2 (*p* = 0.001) when reading the article.

Experienced surgeons (>5 surgeries performed) significantly increased their confidence only by watching the video (8.7–9.5, *p* = 0.042), but did not improve by reading the manuscript (8.5–9.1, *p* = 0.0918).

## Discussion

### Earlier evidence on manuscript vs. video learning

In the last few years, there have been quite a few studies looking at the use of videos as a novel way of teaching surgeries, with overall favorable results. The results have reported less need for staff takeover and fewer errors by surgery residents (4, 5), and better overall understanding and more interest in surgical procedures in medical students (6). One study reported that video was the educational method of choice for residents (7). However, no study reports on the efficiency or efficacy of video-based education compared with learning by reading a manuscript in which the surgical technique is explained.

### Interpretation of pre-intervention confidence

The pre-intervention confidence in both groups was comparable (6.6 in the video group, 6.4 in the manuscript group). These finding are in the range of what one might expect of the confidence of a group of pediatric surgeons in a specific surgical technique.

### Interpretation of post-intervention confidence

As expected, both groups significantly improved their confidence in performing this particular operation. When looking at the overall confidence, the video group and the manuscript group improved similarly: from 6.6 to 7.7 and 6.4 to 7.9, respectively. However, when looking at the six questions separately, the surgeons who read the manuscript improved their confidence significantly on five of six questions, whereas surgeons who watched the video improved only on two questions. One could therefore argue that the manuscript seems better at improving the broader confidence of every aspect of diagnosing and treating rectal atresia and stenosis.

### Interpretation of subgroup analysis

Attending surgeons benefited from both the video and the manuscript, whereas trainees benefitted only by watching the video. It is conceivable that with more general surgery knowledge (anatomy, techniques), it is easier to learn from an article. On the other hand, a trainee with fewer skills benefits from the visual instruction that a video offers. An earlier study found that faculty can name more laparoscopic facts when shown a video than can residents shown the same video, but the study did not measure actual proficiency in surgery or confidence to perform the operation (8).

Interestingly, when categorizing participants according to the number of times they have performed the operation, surgeons with the most experience do not significantly improve their scores when reading the manuscript vs. watching the video. Participants with no experience in this particular surgery at all are better off reading the manuscript.

## Conclusion

Watching a video and reading a manuscript about a specific surgical technique similarly improve a surgeon's confidence in performing the operative technique. However, reading a manuscript improves almost every aspect of the surgery, whereas watching a video does not. Surgeons with no experience seem to win greater confidence in performing a procedure after watching a video, but an actual performance on the procedure itself, and its outcomes can't be concluded from this data.

## Author contributions

CR and AD had the idea, planned and performed the study. VL edited the video and manuscript used for the study. JC did the statistical analysis on the paper. RW and ML edited and corrected the manuscript.

### Conflict of interest statement

The authors declare that the research was conducted in the absence of any commercial or financial relationships that could be construed as a potential conflict of interest.
